# Supplementary material for: Circulating Tumor Cells Develop Resistance to TRAIL-Induced Apoptosis Through Autophagic Removal of Death Receptor 5: Evidence from an In Vitro Model
Source: Cancers (Basel). 2019 Jan 15;11(1):94. doi: 10.3390/cancers11010094 (PMC6356356; doi:10.3390/cancers11010094)
Supplement: Supplementary file 1 [file cancers-11-00094-s001.pdf]

Supplementary Material: Circulating Tumor Cells Develop Resistance to TRAIL-Induced Apoptosis Through Autophagic Removal of Death Receptor 5: Evidence from an In Vitro Model

Julianne D. Twomey and Baolin Zhang

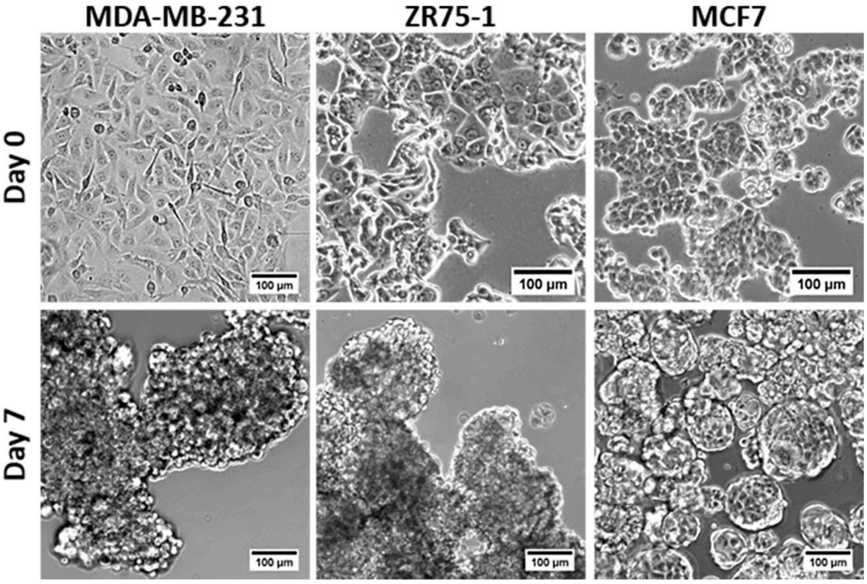

Figure S1. Bright-field microscopy images taken at monolayer (day 0) or following seven days of suspension culture. Taken at 10× magnification, scale bar is 100 µm.

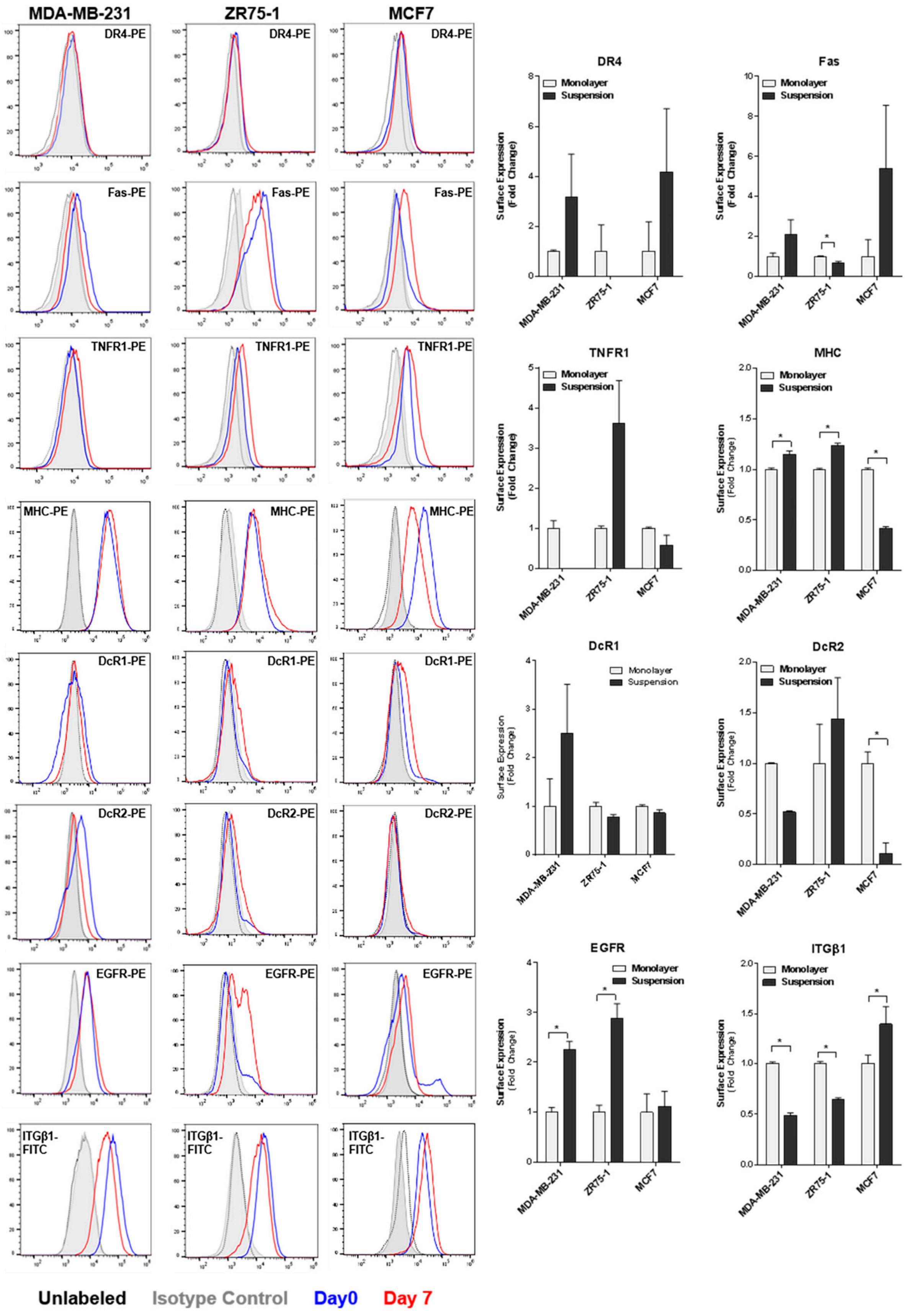

Figure S2. Flow cytometry detection of death receptors and cell surface proteins. Surface expression of DR4, Fas, TNFR1, MHC, DcR1, DcR2, EGFR, and ITGβ1 was analyzed by flow cytometry on BCCs cultured in monolayer and suspension for seven days. Surface expression was analyzed using

Relative Median Fluorescence Intensity (RMFI) of receptors normalized to the corresponding MFI obtained for monolayer cells (Day 0) (mean ± SEM; * $p < 0.05$; $n = 3$).

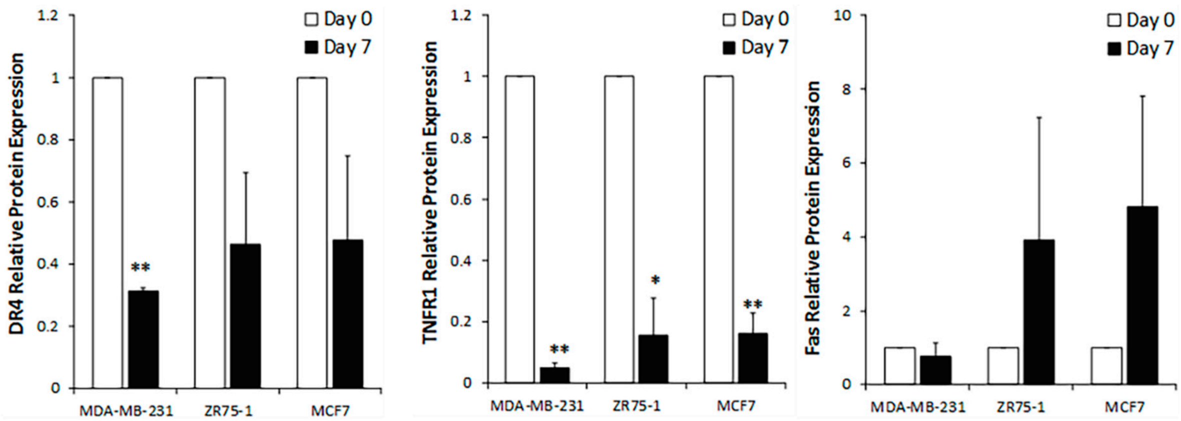

Figure S3. Quantification of death receptor protein expression. Western blot quantification of BCC lines cultured in suspension condition and collected each day. Relative protein expression of DR4, TNFR1, and Fas (relative densitometric analysis) to monolayer (day 0). (* $p < 0.05$, ** $p < 0.01$ to monolayer; $n = 3$).

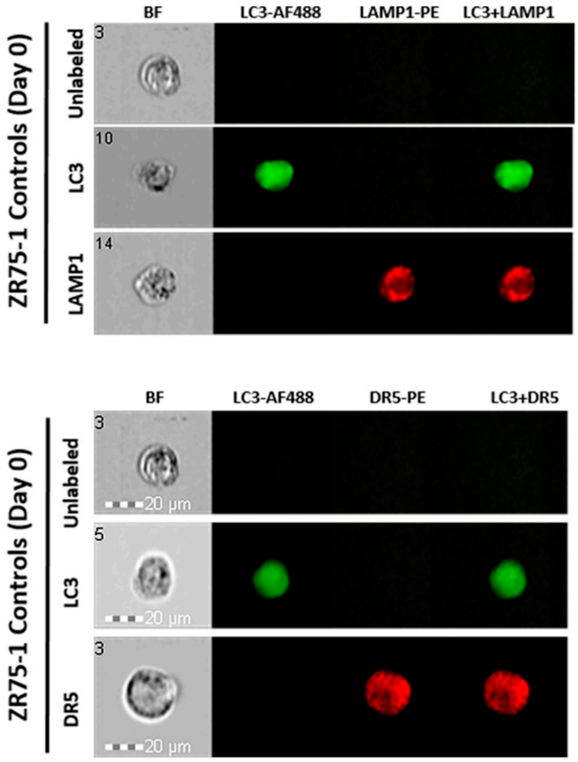

Figure S4. MIFC single fluorophore controls. Images of single fluorophore controls of ZR75-1 cells cultured in monolayer or suspension for 3 or 7 days captured using imaging flow cytometry. Shown are brightfield (BF), LC3-AF488 (green), LAMP1-PE (red) or DR5-PE (red) and a composite image.

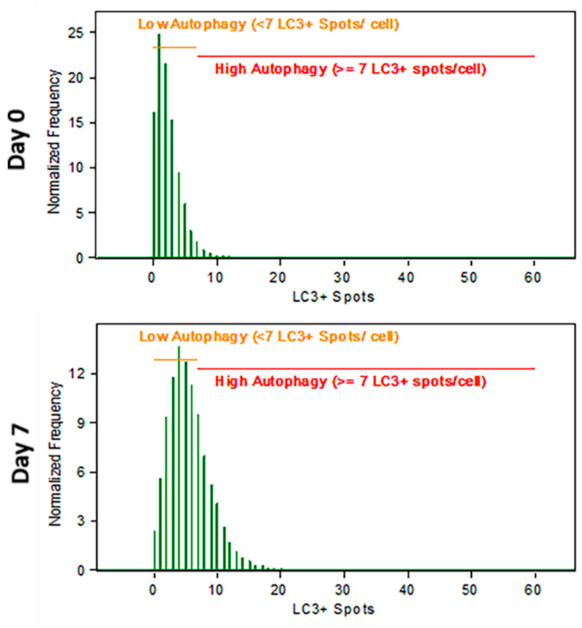

Figure S5. Representative MIFC spot count histograms for LC3-AF488 (autophagosomes). ZR75-1 cells were cultured in monolayer (day 0) and suspension culture for 7 days (day 7) and quantified for LC3 puncta formation. A baseline of 7 LC3 positive puncta was determined from the monolayer cultured cells.

© 2019 by the authors. Licensee MDPI, Basel, Switzerland. This article is an open access article distributed under the terms and conditions of the Creative Commons Attribution (CC BY) license (http://creativecommons.org/licenses/by/4.0/).
